# Zearalenone Biodegradation by the Combination of Probiotics with Cell-Free Extracts of *Aspergillus oryzae* and Its Mycotoxin-Alleviating Effect on Pig Production Performance

**DOI:** 10.3390/toxins11100552

**Published:** 2019-09-20

**Authors:** Chaoqi Liu, Juan Chang, Ping Wang, Qingqiang Yin, Weiwei Huang, Xiaowei Dang, Fushan Lu, Tianzeng Gao

**Affiliations:** 1 College of Animal Science and Veterinary Medicine, Henan Agricultural University, Zhengzhou 450002, China; liuchaoqi2018@stu.henau.edu.cn (C.L.); changjuan2000@henau.edu.cn (J.C.); wangping@henau.edu.cn (P.W.); hww5501@stu.henau.edu.cn (W.H.); 2 Henan Delin Biological Product Co. Ltd., Xinxiang 453000, China; hndlbio@hndlbio.com; 3 Henan Puai Feed Co. Ltd., Zhoukou 466000, China; lufushan@puaifeed.com; 4 Henan Guangan Biotechnology Co., Ltd., Zhengzhou 450001, China; gaotianzeng@groundgroup.com

**Keywords:** Zearalenone, biodegradation, probiotics, cell-free extracts of *Aspergillus oryzae*, pig production performance

## Abstract

In order to remove zearalenone (ZEA) detriment—*Bacillus subtilis*, *Candida utilis*, and cell-free extracts from *Aspergillus oryzae* were used to degrade ZEA in this study. The orthogonal experiment in vitro showed that the ZEA degradation rate was 92.27% (*p* < 0.05) under the conditions that *Candida utilis*, *Bacillus subtilis* SP1, and *Bacillus subtilis* SP2 were mixed together at 0.5%, 1.0%, and 1.0%. When cell-free extracts from *Aspergillus oryzae* were combined with the above probiotics at a ratio of 2:1 to make mycotoxin-biodegradation preparation (MBP), the ZEA degradation rate reached 95.15% (*p* < 0.05). In order to further investigate the MBP effect on relieving the negative impact of ZEA for pig production performance, 120 young pigs were randomly divided into 5 groups, with 3 replicates in each group and 8 pigs for each replicate. Group A was given the basal diet with 86.19 μg/kg ZEA; group B contained 300 μg/kg ZEA without MBP addition; and groups C, D, and E contained 300 μg/kg ZEA added with 0.05%, 0.10%, and 0.15% MBP, respectively. The results showed that MBP addition was able to keep gut microbiota stable. ZEA concentrations in jejunal contents in groups A and D were 89.47% and 80.07% lower than that in group B (*p* < 0.05), indicating that MBP was effective in ZEA biodegradation. In addition, MBP had no significant effect on pig growth, nutrient digestibility, and the relative mRNA abundance of estrogen receptor alpha (*ERα*) genes in ovaries and the uterus (*p* > 0.05).

## 1. Introduction

Zearalenone (ZEA) is one of the nonsteroidal estrogenic mycotoxins produced by *Fusarium* species to cause reproduction disorders in female animals such as abortion, false estrus, and so on [1]. Some of ZEA derivatives competitively bind estrogen receptors in the uterus and ovaries for reducing the ability of estrogen to bind receptors [2,3]. Long-term exposure to low doses of ZEA could alter estrogen receptor beta genes and induce epigenetic modification to inhibit the development of the ovary [4]. However, the relative transactivation activity of ZEA for estrogen receptor alpha was higher than estrogen receptor beta [5]. In sorghum, maize, wheat, rice, barley, and other crops and their sideline products, ZEA can be detected in their natural state during storage, transportation, and processing [6]. It was reported that ZEA was present in 92% of maize, 88% of maize silage, and 97% of small grains samples with a range of 141.30–253.07 μg/Kg [7,8,9]. Another report showed that the incidences and maximal levels of ZEA in raw cereal grains were 46% and 3049 μg/kg respectively, according to the global occurrence data reported during the past 10 years [10]. Generally, pigs are more sensitive to ZEA than other animals. Therefore, it is necessary to take some methods to eliminate ZEA in the animal feeding process, especially for female pigs.

In order to reduce ZEA detriment, many countries and organizations in the world have limited the maximal ZEA contents in food, feed, and cereals. For example, Australia allows a maximal ZEA content of 50 μg/Kg in cereals. ZEA content in cereals and cereal products is not allowed to exceed 100 μg/Kg in Italy. The maximal ZEA content in vegetable oil and cereals is 200 μg/Kg in France [11]. The maximal ZEA content in swine diets is 250 μg/Kg in China and Europe. Even so, ZEA content often exceeds these thresholds because of the different environments and inadequate storage conditions.

In order to detoxify ZEA, some physical, chemical, and biological methods have been conducted [12,13,14,15]. Some reports have shown that ZEA biological degradation is more effective than other methods [16,17]. There were some reports about ZEA biodegradation by microorganisms including *Saccharomyces cerevisiae* [18], *Bacillus subtilis* (*B. subtilis*) ANSB01G [19], and *Lactobacillus plantarum* [20]. In this study, *B. subtilis* SP1, *B. subtilis* SP2, *Candida utilis* (*C. utilis*), and cell-free extracts of *Aspergillus oryzae* (*A. oryzae*) were selected and combined together to determine the effect on ZEA detoxification and pig production performance, which will supply a new feed additive for safe animal feeding and production.

## 2. Results

### 2.1. The Probiotics for ZEA Degradation

In order to obtain the optimal ratio of probiotics for ZEA degradation, an orthogonal design was used in this experiment. Table 1 indicates that the biggest ZEA degradation rate in the nine treatments was 92.27% (*p* < 0.05) under the conditions that *Candida utilis*, *Bacillus subtilis* SP1, and *Bacillus subtilis* SP2 were mixed together at a ratio of A_1_B_2_C_2_ (i.e., 0.5%, 1.0%, and 1.0%, respectively). The analysis showed that the optimal addition ratio of *C. utilis*, *B. subtilis* SP1, and *B. subtilis* SP2 was A_1_B_1_C_3_ (i.e., 0.50%, 0.50%, and 1.50%). However, further ZEA degradation experiments indicated that A_1_B_2_C_2_ was better than A_1_B_1_C_3_. Table 2 indicated that that the constructed model was accurate and acceptable (*p* < 0.01). Among the three strains of microbes, *C. utilis* had a significant effect on the model for ZEA degradation (*p* < 0.01), followed by *B. subtilis* SP1 *and B. subtilis* SP2 (*p* > 0.05).

### 2.2. ZEA Degradation by the Combined Probiotics and Cell-Free Extracts of A. oryzae

**Figure 1:**
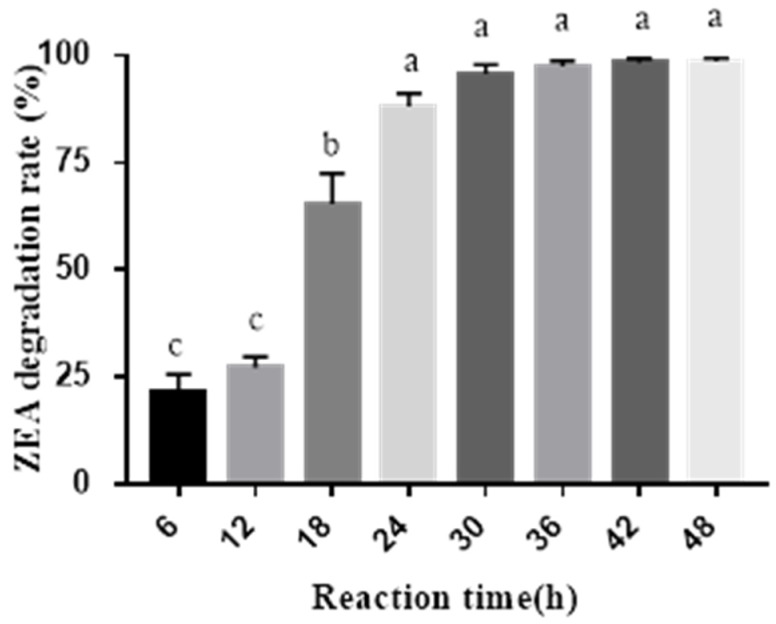
ZEA degradation by cell-free extracts of *A. oryzae* at different reaction times. Note: Data with the same lowercase letters in the bars are insignificantly different from each other (*p* > 0.05); while data with different lowercase letters in the bars are significantly different from each other (*p* < 0.05).

For measuring the effect of combined probiotics with cell-free extracts of *A. oryzae* on ZEA degradation, the effectiveness of extracts from *A. oryzae* was first determined. Figure 1 indicates that the ZEA degradation rate was 88.16% after 24 h enzymatic hydrolysis (*p* < 0.05); thereafter, there was no significant difference among the different groups (*p* > 0.05), even though ZEA degradation rate reached 98.62% after 48 h enzymatic reaction. Table 3 indicated that the best ZEA degradation rate was 95.15% when the ratio between probiotics and cell-free extracts of *A. oryzae* was 2:1 (*p* < 0.05). According to this ratio, mycotoxin-biodegradation preparation (MBP) was made for the further pig feeding experiment. ZEA degradation rate of individual probiotics was lower than both combinations of probiotics and cell-free extracts of *A. oryzae* (*p* < 0.05), indicating the effectiveness of combination; however, there was no significant difference between single cell-free extracts and both combinations for ZEA degradation (*p* > 0.05).

### 2.3. Effect of MBP on Pig Growth Performance and Nutrient Digestibility

In order to determine the effect of MBP on alleviating ZEA for pig production performance, feeding experiments were conducted. Table 4 indicates that there were no significant differences in average daily gain (ADG), average daily feed intake (ADFI), feed conversion rate (F/G), and digestibility of crude protein (CP), crude fat (CF), phosphorus (P), and calcium (Ca) among the 5 groups (*p* > 0.05). However, it showed that the above parameters could be improved by MBP addition in groups D and E, compared to group B.

### 2.4. Pig Gut Microbiota Affected by MBP

**Figure 2:**
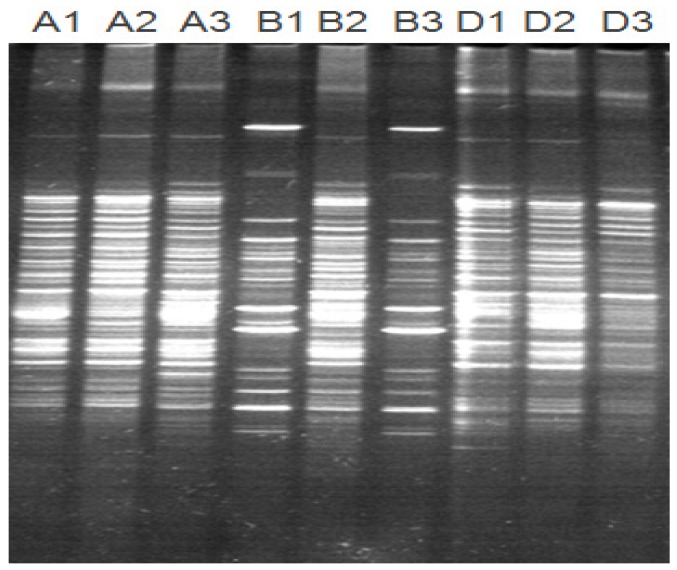
The electrophoresis diagram of DGGE in gilt large intestines. Note: Lanes A1–A3, contents of large intestine in group A; Lanes B1–B3, contents of large intestine in group B; Lanes D1–D3, contents of large intestine in group D.

**Figure 3:**
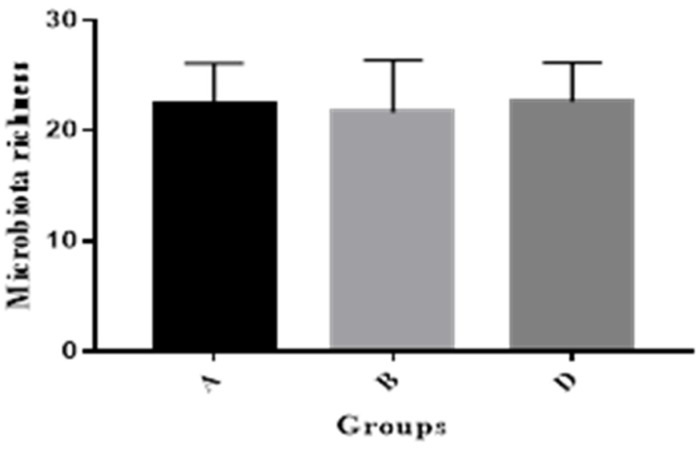
Microbial richness calculated by the number of bands in the electrophoresis diagram of DGGE.

**Figure 4:**
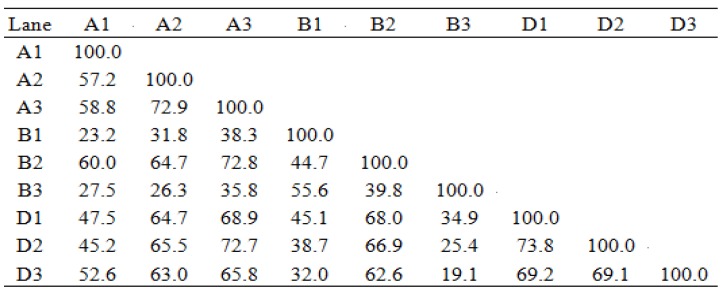
The microbial similarity coefficients in different samples.

The DGGE profile can reflect the structure of the gut bacterial community, and the number of bands indicates the richness of phylogenetic types. Figure 2 indicates that the numbers of discernible microbial bands in gilt large intestines were 18, 24, and 25 in group A; 18, 20, and 27 in group B; and 19, 23, and 26 in group D. The universal bands were 14, 13, and 13 in groups A, B, and D, respectively. The sizes of bands were about 220 bp. After statistical analysis of the bands, there was no significant difference in microbial richness among the three groups in Figure 3. The gut microbial similarity coefficient analysis in Figure 4 showed that similarity coefficients were 57.2%–72.9% in group A, 38.3%–72.8% in group B, and 69.1%–73.8% in group D, indicating that the gut microbiota was more stable by MBP addition in group D. 

### 2.5. The Vulvar Area and Serum Parameters

**Figure 5:**
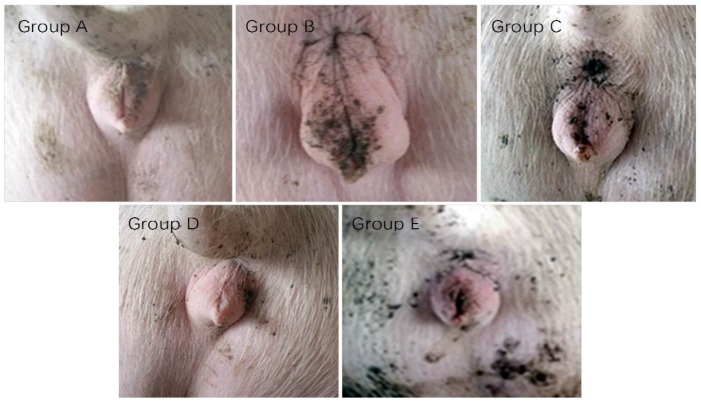
Effect of MBP on gilt vulvar representation.

Gilt vulvar area can reflect the effect of ZEA on female pig reproduction status. The effect of MBP on alleviating gilt vulvar area caused by ZEA in Figure 5 showed that, during the first 15 d, the vulvar area in group A increased slightly, while the other groups increased greatly, especially for group B. Fifteen days later, the vulvar area in each group increased slightly, and the order of vulvar area was: group B > group C > groups D and E > group A, indicating the effectiveness of MBP for weakening ZEA detriments.

In addition, ZEA or MBP had no significant effect on serum biochemical parameters such as urea nitrogen (UN), total cholesterol (TC), glucose (GLU), triglyceride, high-density lipoprotein (HDL), low-density lipoprotein (LDL), total protein (TP), albumin (ALB), globulin (GLO), albumin and globulin ratio (A/G), alanine aminotransferase (ALT), aspartate aminotransferase (AST), alkaline phosphatase (ALP), immunoglobulin G (IgG), immunoglobulin M (IgM), glutathione peroxidase (GSH-Px), and estradiol (E_2_). It can be concluded that ZEA or MBP could not cause significant effects on the normal physiological and metabolic status of pigs in this study.

### 2.6. The Relative Organ Weight and ERα mRNA Abundance in Ovaries and the Uterus

ZEA may influence the relative organ weight and ERα mRNA abundance for female pigs. However, Table 5 shows that there was no significant difference for relative organ weight and ERα mRNA abundance in ovaries and the uterus among groups A, B, and D (*p* > 0.05), which showed that 300.00 μg/kg ZEA in pig diets was not enough to cause significant changes in relative organ weight and ERα mRNA abundance in ovaries and the uterus, even though MBP addition could alleviate ZEA hazards to some extent.

### 2.7. ZEA Concentrations in Serum, Relative Tissues, and Gut

In order to realize the status of ZEA metabolism and deposit for pigs, the ZEA distribution was measured. Table 6 shows that residual ZEA in serum, longissimus dorsi, uterus, and liver in groups A, B, and D was not detected. ZEA concentrations in jejunal contents in groups A and D were 89.47% and 80.07% lower than that in group B (*p* < 0.05). ZEA concentrations in the large intestine in groups A and D were 68.57% (*p* < 0.05) and 12.79% (*p* > 0.05) lower than that in group B, respectively, indicating the effectiveness of MBP for ZEA degradation.

## 3. Discussion

As one of the most ubiquitous mycotoxins, ZEA has caused serious harm to animals and humans and has led to a great waste of food resources every year [21]. There are two ways to solve this problem. One way is to inhibit ZEA production from microbes, which is hard to conduct due to the difficult control of ZEA-excreting microbes in the environment; another way is to eliminate ZEA harm to animals and humans by chemical, physical, and biodegradable methods [12,13,14,15]. Although physical and chemical methods can eliminate ZEA harm, they also reduce the nutritional value of food and pollute the environment. Many researchers have indicated that ZEA biodegradation or absorption by microbes and mycotoxin-degrading enzymes is the most effective method [16,17,22,23].

It was found that the peroxiredoxin gene from *Acinetobacter* sp. SM04 was cloned in *Escherichia coli* BL21 to excrete one recombinant protein for ZEA detoxification [24]. *Saccharomyces cerevisiae* and *B. subtilis* have been found to be able to degrade ZEA around 90% [25,26]. *Broomyces pink* and *B. subtilis* were reported to open the ZEA lactone ring to form nontoxic compounds by hydrolyzing lactone bonds [27,28]. In order to increase the ZEA degradation rate, two strains of *B. subtilis* and one strain of *C. utilis* with good ZEA degradation abilities have been selected and combined with ZEA degradation enzymes from *A. oryzae* in this study. 

*A. oryzae* is one kind of microbe that produces complex enzymes, which will help to increase nutrient availability and degrade mycotoxins. It was found that *A. oryzae* could convert ZEA into other metabolites and reduce the toxicity of toxins [29]. Another report showed that the deoxynivalenol degradation rate was over 92% after *A. oryzae* was inoculated with corn culture medium for 21 d [30]. In this research, cell-free extracts of *A. oryzae* could degrade ZEA by 98.62% after 48 h enzymatic reaction, which was better than the previous report. When cell-free extracts of *A. oryzae* were combined with the probiotics, the ZEA degradation rate was higher than the individual, indicating that there was a good cooperation between them. This result corresponds with the previous report for aflatoxin removal [31]. Generally, the probiotics are able to regulate gastrointestinal microbiota for animal health, except for its ZEA-removing ability. Therefore, the combination of beneficial bacteria and *A. oryzae* extracts will have great applications in the fields of animal production and food and feed processing.

ZEA is widely distributed in all kinds of grain crops and feedstuffs. The prevention and removal of ZEA pollution of grain has become a hotspot for researchers. It was found that the growth performance of weaned piglets fed with 1000 μg/Kg ZEA diet for 24 d and the young gilts fed with 1.5–2.0 mg/kg ZEA diet for 28 d had no significant changes [32,33]. In this study, after feeding with 300 μg/Kg ZEA diet for 60 d, ADG, ADFI, F/G, and nutrient digestibility of young pigs had no significant difference regardless of MBP addition or not. It is possible that this concentration of ZEA added in the diet was not enough to retard pig growth even though it may influence reproduction for female pigs.

This study showed that MBP addition was able to increase gut microbial similarity coefficients to help maintain gut microbial balance for pig health, in agreement with the former research, in which it was reported that *Saccharomyces cerevisiae* subsp. *boulardii* strain had the potential as feed additives to modulate bacterial populations associated with gut health for piglets [34]. 

After the diets contaminated with ZEA are fed to animals, ZEA will have three metabolic pathways: (1) ZEA is absorbed in the gut and remains in the animal’s body; (2) it is degraded by the gut microbes and enzymes; and (3) some ZEA will be discharged with the feces. It was found that the residual amount of ZEA in gilt livers increased significantly when dietary ZEA contents were 500 or 2000 μg/Kg [35]. However, ZEA was not detected in serum, longissimus dorsi, the uterus, and liver in this study, inconsistent with the above. This may be due to the low ZEA content (300 μg/Kg) in pig diets. This research showed that ZEA concentrations in jejunal contents in group B was significantly higher than that in group A and group D, indicating that part of ZEA was eliminated by MBP in the gastrointestinal tract, which proved the effectiveness of MBP for degrading ZEA in pig guts.

Serum ALT, AST, and ALP are important indicators to measure the degree of liver lesions. Serum IgG and IgM are the main factors that participate in the humoral immune response. Their normal levels in this study indicated that liver cells and immune systems have not been damaged by ZEA at 300 μg/Kg dosage. The previous report showed that long-term exposure to a low dose of ZEA had no significant negative effect on serum ALT, AST, ALP, and E2 levels in pigs [36], which is in agreement with this study. The organ indexes of heart, liver, kidney, and spleen were not significantly changed by feeding the weaned piglets with 316 μg/Kg ZEA diets [37], which corresponds with this study. The reason may be due to the low ZEA dose in pig diets. 

The chemical structure of ZEA is the same as estrogen; therefore, ZEA can competitively bind estrogen receptors to cause reproduction disorders, in which the red and swelling vulva of female pig is the common apparent symptom. This research showed that 300 μg/Kg ZEA could cause a red and swelling vulva; however, MBP addition was able to alleviate the symptoms, in agreement with the previous research with *Bacillus* addition [38]. It was found that MBP was better than only *Bacillus subtilis* for degrading ZEA and keeping the microbiota stable in the pig gut [38].

It was found that the relative expression of estrogen receptor gene ERα mRNA in the ZEA group was significantly higher than that in control group [39]. Another research study showed that the serum E2 of females was significantly decreased by the addition of 1.05 mg/kg ZEA in diets [32]. Generally, ZEA could cause DNA double-strand breaks and affect the proliferation of granulosa cells, and the addition of an estrogen receptor antagonist could improve this symptom, indicating that ZEA could damage granulosa cells through the estrogen receptor pathway [40]. It was reported that the relative expression levels of ERα genes in brain, liver, and gonads were not significantly affected by low doses of ZEA, but they were significantly affected by high doses of ZEA. This process changed the HPG axis by altering gene expression of the steroid hormone-encoding gene to affect reproductive function [41]. In this study, the relative expression of estrogen receptor gene ERα mRNA in ovaries and the uterus was not significantly different among the three groups, which does not correspond with the above results. The reason may be due to the low levels of ZEA in the diet in this study.

## 4. Conclusions

The combination of beneficial microbes (*B. subtilis* SP1, *B. subtilis* SP2, *C. utilis*) and cell-free extracts from *A. oryzae* could degrade ZEA effectively in vitro. MBP addition could alleviate ZEA negative effects in gilts by decreasing vulvar swelling, improving ZEA degradation in the jejunum, and keeping normal growth performance and gut microbiota stable.

## 5. Materials and Methods

### 5.1. Probiotics and Experimental Materials

*B. subtilis* SP1, *B. subtilis* SP2, *C. utilis,* and *A. oryzae* with high ZEA-degrading ability were purchased from China General Microbiological Culture Collection Center (CGMCC, Beijing, China). ZEA was purchased from Sigma-Aldrich (St. Louis, MO., USA) and diluted in methanol as stock solution (100 μg/mL). PBS buffer was prepared according to the previous protocol [42]. The compositions of media for *B. subtilis*, *C. utilis,* and *A. oryzae* incubations were prepared according to the published protocols [43,44]. Then, three kinds of probiotics were harvested and stored at 4 °C.

### 5.2. Degradation of ZEA by Probiotic Incubation

The visible counts of three kinds of microbes were adjusted to 1.0 × 10^8^ CFU/mL, respectively. The orthogonal design (5 mL reaction system) with three factors (*B. subtilis* SP1, *B. subtilis* SP2, and *C. utilis*) and three levels (25, 50, and 75 μL) were used to investigate the effect of beneficial microbes on degrading ZEA. Each group contained 1 μg/mL ZEA. The contents of ZEA in the samples were determined by enzyme-linked immunosorbent assay (ELISA) according to Huang’s report [44], which was highly consistent with high-performance liquid chromatography (HPLC). ZEA degradation rates were calculated according to the following formula: ZEA degradation rate = (1 − ZEA concentration in treatment/ZEA concentration in control) × 100%. All experiments were conducted in triplicate.

### 5.3. Preparation of Cell-Free Extracts from A. oryzae

*A. oryzae* was inoculated in solid medium at 30 °C for 3 d. After incubation, 15 g solid incubation was mixed with 300 mL physiological saline and put in a rotary shaker at 180 rpm for 2 h. The mixture was filtered with eight-layer gauze, centrifuged at 10,000 g for 10 min, passed through Whatman No.4 filter paper (20 to 25 µm pore diameters), and filtered with 0.22 μm Minisart High-flow filter (Sartorius Stedim Biotech Gmbh, Goettingen, Germany). Finally, it was stored at 4 °C for further use.

The final volumes of 60 mL filtrates with an initial ZEA concentration of 1 μg/mL in 250 mL conical flasks were incubated at 37 °C in a rotary shaker at 180 rpm, and the samples were collected after 0, 6, 12, 18, 24, 30, 36, 42, and 48 h incubation, respectively. The ZEA degradation rates were measured at different times. The ZEA degradation activity (31.0 U/L) from cell-free extracts of *A. oryzae* was determined with the previous protocol [42] and modified as the following: the amount of enzyme that could degrade 1 ng ZEA per min at pH 7.0 and 37 °C was defined as one unit.

### 5.4. ZEA Degradation by Probiotics Combined with Cell-Free Extracts of A. oryzae

The probiotics for degrading ZEA were added in YPD medium at the above ratio. The probiotics and cell-free extracts from *A. oryzae* were mixed at the volume ratios of 1:1, 1:2, 1:3, 2:1, 2:3, 3:1, 3:2, 1:0, and 0:1 as treatment groups. The final volume of the reaction system was adjusted to 3 mL with YPD medium. All the groups received 30 μL ZEA (100 μg/mL), were incubated at 37 °C in a rotary shaker at 180 rpm for 48 h, and were finally put in boiling water for 30 min to terminate the reaction. 

### 5.5. Animals and Management 

A total of 120 young pigs (Duroc × Landrace × Yorkshire) at the ages of 78–84 d were randomly divided into 5 groups, with 3 replicates in each group and 8 pigs (half male and half female) in each replicate. All animals used in this experiment were managed according to the guidelines of Animal Care and Use Ethics Committee in Henan Agricultural University (SKLAB-B-2010-003-01). The pigs were provided with a corn/soybean basal diet formulated to meet pig nutrient requirements according to the NRC (2012) [45]. The diets and water were provided ad libitum. The total experimental period was 60 d. After the feeding experiment, three gilts from groups A, B, and D were slaughtered for further analyses, respectively. Body weight and feed intake were recorded. ZEA used in animal feeding experiments was purchased from Wuhan 3B Scientific Corporation (Wuhan, China). The experimental designs were as follows:

Group A: Basal diet with 86.19 μg/kg ZEA
Group B: Basal diet containing 300.00 μg/kg ZEA
Group C: Basal diet containing 300.00 μg/kg ZEA and 0.05% MBP
Group D: Basal diet containing 300.00 μg/kg ZEA and 0.10% MBP
Group E: Basal diet containing 300.00 μg/kg ZEA and 0.15% MBP

### 5.6. Nutrient Digestibility Measurement

Fecal samples were taken without contamination from each of 5 pigs in each group for 3 d at the end of the experiment. The individual fecal sample was mixed, selected, and stored at −20 and 4 °C, respectively. The fecal samples stored at −20 °C were dried to determine nutrient digestibility. CP, CF, Ca, and P in diets and feces were measured with Kjeldahl, ether extract, potassium permanganate (KMnO_4_), and ammonium molybdate ((NH_4_)_6_Mo_7_O_24_) protocols, respectively [46]. The insoluble ash of hydrochloric acid in feed and feces was used as an indicator to calculate the nutrient digestibility with the following formula: Nutrient digestibility = (nutrient content in diets − nutrient content in feces)/nutrient content in diets × 100%.

### 5.7. Vulvar Area Measurement of Gilts

From the beginning of the experiment, vulvar areas of gilts were observed every day, and six gilts in each group were selected and measured at 15, 30, 45, and 60 d. The measurement method was based on a previous report, the vulvar area = π × vulvar width × vulvar length/4 [38].

### 5.8. Serum Parameter Determination

Blood samples were taken from the anterior vena cava of three pigs in each group. After the blood was tilted at room temperature for 3 h, the serum was collected by Transferpettor and stored in a centrifuge tube at −20 °C for further analysis. The serum biochemical parameters such as UN, TC, GLU, triglycerides, HDL, LDL, TP, ALB, GLO, A/G, ALT, AST, ALP, IgG, and IgM were measured with a 7600-020 Automatic Analyzer (Hitachi Ltd., Tokyo, Japan) in the Biochemical Laboratory of Zhengzhou University, Zhengzhou, China. The concentrations of GSH-Px and E_2_ were respectively measured by ELISA quantification kits (Nanjing Jiancheng Bioengineering Institute, Nanjing, China).

### 5.9. Relative Organ Weight and ERα mRNA Abundance in Ovaries and the Uterus

After the feeding experiment, 3 gilts from 3 representative groups (groups A, B, and D) were selected and slaughtered, respectively. The gut contents were taken and frozen for further gut microbiota analysis, and the organs were taken and weighed. The formula for calculating the organ index was as follows: organ index = organ weight (g)/living weight (Kg). Some parts of ovaries and the uterus were stored in liquid nitrogen for mRNA abundance analyses.

In order to measure the expression levels of ERα in ovaries and the uterus, the total RNA was extracted by using RNAiso Plus kits (TaKaRa, Dalian, China). The result was checked with 1% agarose gel electrophoresis. The reaction system for cDNA consisted of 2 μL 4 × gDNA wiper Mix, 4 μL RNA template, and 2 μL RNase-free water. It was kept at 42 °C for 2 min, mixed with 2 μL 5×HiScript^®^IIqRT SuperMixII (Vazyme Biotech Co., Ltd., Nanjing, China), and then kept at 25 °C for 10 min, 50 °C for 30 min, and 85 °C for 5 min. The cDNA samples were stored at −20 °C for further use. The qPCR reaction consisted of 10.0 μL AceQ^®^ qPCR SYBR^®^ Green Master Mix, 0.4 μL ROX, 0.4 μL Primer F (10 μM), 0.4 μL Primer R (10 μM), 2.0 μL cDNA, and 6.8 μL RNase-free water (Vazyme Biotech Co., Ltd., Nanjing, China). The primers for ERα and glyceraldehyde-3-phosphate dehydrogenase (GAPDH) genes were as follows: Forward 5′-GACAGGAACCAGGGCAAGT-3′, Reverse 5′-ATGATGGATT TGAGGCACAC-3′ for ERα; Forward 5′-ATGGTGAAGGTCGGAGTGAA-3′, Reverse 5′-CGTGGGTGGAATCATACTGG-3′ for GAPDH. The thermal program of qPCR consisted of 1 cycle at 95 °C for 10 min, 40 cycles of 95 °C for 15 s, 57 °C for 30 s, and then stored at 37 °C for 30 s. Gene-specific amplification was determined by a melting curve analysis and agarose gel electrophoresis. The cycle threshold value was analyzed (iQ5 detection System) and transformed to a relative quantity using the 2^−ΔΔCT^ method with the highest quantity scaled to 1 [47]. *GAPDH* gene was used as the reference gene for stability of expression to standardize the relative expression of the gene investigated.

### 5.10. DNA Extraction of Gut Bacteria and PCR-DGGE

About 1.0 g of gut sample from the large intestine was defrosted, put into a 10 mL centrifuge tube, washed with 5 mL PBS, and centrifuged at 500 *g* at 4 °C for 5 min to collect the supernatant according to the previous protocol [48]. The washing step was repeated 3 times. The supernatants from 3 washing steps were mixed together and centrifuged at 8000 *g* for 5 min to collect the pellet. The bacterial DNA was extracted with MiniBEST Bacterial Genomic DNA Extraction Kit (TaKaRa, Dalian, China), dissolved in 100 μL TE, and stored at −20 °C as the template for PCR amplification.

The primers of V3 regions of bacterial 16S rRNA genes [49] were F341: CGCCCGCCGCGCGCGGCGGGCGGGGCGGGGGCACGGGGGGCC TACGGGAGGCAGCAG, and R518: ATTACCGCGGCTGCTGG. The PCR system (50 μL) consisted of 25 μL Taq Master Mix, 5 μL extracted gut DNA, 1 μL primer F341-GC (10 μM), 1 μL R518 (10 μM), and 18 μL RNase-free water (Beijing Comwin Biotech Co., Ltd. Beijing, China). The program consisted of initial DNA denaturation at 95 °C for 5 min, 35 cycles of 95 °C for 30 s, 55 °C for 30 s, 72 °C for 40 s, and a final extension at 72 °C for 10 min. The PCR products were detected by 1.5% agarose gel electrophoresis (Beijing Solarbio Science & Technology Co., Ltd., Beijing, China).

The PCR products were separated in 8% polyacrylamide gel containing a 35% to 60% gradient of urea and formamide increasing in the direction of electrophoresis. Electrophoresis was initiated by prerunning at a voltage of 200 V for 10 min and then at a constant voltage of 90 V for 14 h at 60 °C. After electrophoresis, the gel was stained with 0.5 μg/mL ethidium bromide for 30 min and then washed with deionized water for 10 min.

DGGE gels were analyzed using the software of Quantity One 4.6.6 (BioRad, California, USA, 2017). Microbial similarity coefficients (SC) between DGGE profiles were determined as follows: SC = 2 × J/(Nx + Ny), where Nx is the number of DGGE bands in lane x, Ny represents the number of DGGE bands in lane y, and J is the number of common DGGE bands [50].

### 5.11. Determination of ZEA Content in Serum and Tissues

Longissimus dorsi, liver, and uterus tissues were ground into powder with liquid nitrogen. A total of 1 mL serum or 5 g samples were added to 25 mL 70% methanol, shaken for 3 min, centrifuged at 10,000 *g* for 5 min, then 1 mL supernatant was mixed with 1 mL deionized water for further determination. The ZEA contents in all samples were measured by RIDASCREEN^®^ FAST ZEA SC test kit (R-Biopharm, Darmstadt, Germany).

### 5.12. Statistical Analyses

The experimental data were analyzed as a single factor design by analysis of variance (ANOVA) using IBM SPSS-Statistics Program 20.0 (IBM, New York, NY, USA, 2012), and they are expressed as means and standard errors (SEs). The means were evaluated with Tukey’s multiple range test, and differences were considered statistically significance at *p* < 0.05.

## Figures and Tables

**Table 1:** Zearalenone (ZEA) degradation rate by probiotics.

Number	*C. utilis* (%) A	*B. subtilis SP1* (%) B	*B. subtilis SP2* (%) C	ZEA Content (μg/L)	ZEA Degradation Rate (%)
1	0.5	0.5	0.5	79.14 ± 24.91e	85.00 ± 4.98ab
2	0.5	1	1	40.80 ± 6.68e	92.27 ± 1.30a
3	0.5	1.5	1.5	40.82 ± 12.25e	92.26 ± 2.45a
4	1	0.5	1	123.53 ± 14.87de	76.58 ± 2.41bc
5	1	1	1.5	163.59 ± 16.09cde	68.99 ± 0.78cd
6	1	1.5	0.5	247.85 ± 42.91c	53.02 ± 3.63e
7	1.5	0.5	1.5	238.45 ± 13.50cd	54.80 ± 7.96e
8	1.5	1	0.5	382.36 ± 102.93b	27.52 ± 13.24f
9	1.5	1.5	1	282.28 ± 20.81bc	46.49 ± 2.89e
Control	0	0	0	527.52 ± 63.55a	
T1	269.2	215.47	166		
T2	198.58	189.18	214.96		
T3	128.48	191.61	215.3		
X1	89.73	71.82	55.33		
X2	66.19	63.06	71.65		
X3	42.83	63.87	71.77		
*R*	46.9	8.76	16.44		
Impact order		A > C > B			
Optimal solution		A_1_B_1_C_3_			

Note: T1, T2, and T3 mean the sums of all ZEA degradation rates at the levels of 0.50%, 1.00%, and 1.50% respectively; X1, X2, and X3 mean the averages of all ZEA degradation rates at the levels of 0.50%, 1.00%, and 1.50% respectively; *R* represents the D-value between the maximum and minimum averages of each factor at different levels, and a bigger *R* value indicates that the factor is more important for a higher ZEA degradation rate. Data with the same lowercase letters in the same columns are insignificantly different from each other (*p* > 0.05); while data with different lowercase letters in the same columns are significantly different from each other (*p* < 0.05); the same as below.

**Table 2:** Main effect analyses among different factors.

Sources	Sum of Squares	DF	Mean Square	*F*	*p*
Corrected Model	2808.72	6	468.12	73.68	0.0132 *
A	2545.95	2	1272.98	200.35	0.0051 *
B	83.97	2	41.99	6.61	0.1312
C	178.79	2	89.40	14.07	0.0661
Error	12.71	2	6.35		
Corrected total	2821.42	8			

Note: “*” shows significant differences.

**Table 3:** ZEA degradation by the combined probiotics with cell-free extracts of *A. oryzae* for a 48 h reaction.

Groups	Probiotics: Cell-free extracts of *A. oryzae*	ZEA Content (μg/L)	ZEA Degradation Rate (%)
1	1:1	120.57 ± 21.56c	89.89 ± 1.81a
2	1:2	128.27 ± 79.74c	89.24 ± 6.69a
3	1:3	64.31 ± 83.73c	94.60 ± 7.02a
4	2:1	57.81 ± 12.83c	95.15 ± 1.08a
5	2:3	108.19 ± 28.55c	90.92 ± 2.39a
6	3:1	84.05 ± 73.25c	92.95 ± 6.14a
7	3:2	102.72 ± 72.17c	91.83 ± 6.05a
8	1:0	349.38 ± 34.13b	70.69 ± 2.86b
9	0:1	170.59 ± 64.49c	85.69 ± 5.41a
Control	0:0	1192.10 ± 86.55a	

Note: Data with the same lowercase letters in the same columns are insignificantly different from each other (*p* > 0.05); while data with different lowercase letters in the same columns are significantly different from each other (*p* < 0.05).

**Table 4:** Effect of mycotoxin-biodegradation preparation (MBP) on growth performance and nutrient digestibility of pigs exposed to ZEA.

Items	Group A	Group B	Group C	Group D	Group E
Initial weight (Kg)	34.83 ± 1.06	34.92 ± 1.34	35.17 ± 2.35	34.88 ± 1.27	34.83 ± 1.89
Final weight (Kg)	90.00 ± 4.23	83.58 ± 4.47	82.63 ± 1.80	88.54 ± 2.47	88.33 ± 3.70
ADG (Kg)	0.9211 ± 0.0812	0.8088 ± 0.0811	0.7886 ± 0.0304	0.8913 ± 0.0412	0.8908 ± 0.0321
ADFI (Kg)	2.612 ± 0.151	2.402 ± 0.171	2.389 ± 0.080	2.614 ± 0.140	2.542 ± 0.079
F/G	2.842 ± 0.100	2.963 ± 0.121	3.019 ± 0.213	2.914 ± 0.020	2.771 ± 0.169
CP digestibility (%)	89.73 ± 0.16	91.97 ± 0.94	89.53 ± 1.67	89.23 ± 1.25	90.67 ± 1.93
CF digestibility (%)	76.99 ± 3.94	80.87 ± 5.56	82.63 ± 4.28	78.41 ± 3.11	75.32 ± 1.22
P digestibility (%)	88.19 ± 0.42	87.67 ± 0.48	86.38 ± 1.58	87.21 ± 0.20	88.98 ± 0.07
Ca digestibility (%)	78.83 ± 0.22	77.51 ± 0.75	77.44 ± 0.09	76.48 ± 1.19	77.21 ± 0.89

Note: Data without lowercase letters in the same rows are insignificantly different from each other (*p* > 0.05). Group A: control; Group B: 300.00 μg/kg ZEA; Groups C, D, and E: 300.00 μg/kg ZEA plus 0.05%, 0.10%, and 0.15% MBP, respectively. Note: average daily gain (ADG), average daily feed intake (ADFI), feed conversion rate (F/G), and digestibility of crude protein (CP) and crude fat (CF).

**Table 5:** Effect of MBP on serum E2 content, relative organ weight, and ERα mRNA in ovaries and the uterus.

Items	Group A	Group B	Group D
Heart (g/Kg)	3.241 ± 0.100	3.177 ± 0.311	3.616 ± 0.194
Liver (g/Kg)	15.97 ± 1.81	16.33 ± 0.80	18.67 ± 1.67
Spleen (g/Kg)	1.365 ± 0.249	1.605 ± 0.041	1.703 ± 0.164
Kidney (g/Kg)	2.960 ± 0.172	2.967 ± 0.221	3.432 ± 0.496
Uterus (g/Kg)	1.245 ± 0.752	1.932 ± 0.587	1.673 ± 0.318
Serum E2 (pg/ml)	109.03 ± 8.29	112.48 ± 15.75	103.88 ± 5.06
ERα mRNA abundance in ovaries	0.7899 ± 0.1021	1.171 ± 0.141	1.171 ± 0.122
ERα mRNA abundance in uterus	1.010 ± 0.086	1.111 ± 0.153	1.071 ± 0.191

Note: Data without lowercase letters in the same rows are insignificantly different from each other (*p* > 0.05).

**Table 6:** Effect of MBP on ZEA concentrations in pig serum, tissues, and gut (µg/Kg).

Items	Group A	Group B	Group D
Serum	—	—	—
Longissimus dorsi	—	—	—
Uterus	—	—	—
Liver	—	—	—
Contents in jejunum	11.64 ± 0.27b	110.54 ± 16.19a	22.03 ± 8.20b
Contents in large intestine	43.45 ± 2.44b	138.23 ± 4.67a	120.55 ± 18.87a

Note: Data with the same lowercase letters in the same rows are insignificantly different from each other (*p* > 0.05); while data with different lowercase letters in the same rows are significantly different from each other (*p* < 0.05). “—” indicates no detection.
